# Development of a hydrological model for vegetated swales under different rainfall and swale properties

**DOI:** 10.1002/wer.70014

**Published:** 2025-02-04

**Authors:** Kebir Emre Saraçoğlu, Cevza Melek Kazezyılmaz‐Alhan

**Affiliations:** ^1^ Biological and Agricultural Engineering Department North Carolina State University Raleigh North Carolina USA; ^2^ Civil Engineering Department Istanbul University‐Cerrahpaşa Istanbul Türkiye

**Keywords:** drainage flow, green infrastructure, hydrological model, Rainfall–Watershed–Swale (RWS), surface runoff, vegetated swale

## Abstract

Global warming and climate change affect the number and magnitude of flash floods dramatically, which necessitate effective and innovative solutions. Low Impact Development (LID) has gained importance recently in decreasing the negative impacts of floods in urban areas. LID such as swale, bioretention, infiltration trench, and rain garden emerge as an alternative or complementary solution to the traditional infrastructure stormwater drainage systems. Vegetated swales are one of the most common types of LID used for stormwater runoff control. Although existing software programs have recently integrated modules for LID into their modeling and design, they need improvement for accurate simulations of the hydrological behavior of vegetated swales. Therefore, in this study, a mathematical model was developed to simulate vegetated swale hydrology. The model calculates the overland flow over the watershed area, the overflow over the swale surface, and the drainage flow at the bottom of the swale. A large‐scale experimental setup called Rainfall–Watershed–Swale (RWS) was employed in developing the mathematical model, and data obtained from the RWS was used to calibrate the model. Results show that the developed hydrological model accurately simulates the hydrological behavior of swales under different rainfall events and swale properties.

**Practitioner Points:**

- A mathematical model was developed to simulate vegetated swale hydrology.
- Experimental data from the rainfall–watershed–swale system was employed for model development and calibration.
- The mathematical model effectively calculates the overflow and drainage flow of a vegetated swale.
- The model provides a practical tool for the design of vegetated swales.

## Graphical abstract

A mathematical model for vegetated swale under different rainfall and swale properties was developed to characterize its hydrological behavior by employing the experimental data of the Rainfall–Watershed–Swale system. The overland flow, overflow, and drainage flow can be calculated by the mathematical model, which provides an effective tool for designing sustainable urban stormwater management solutions.

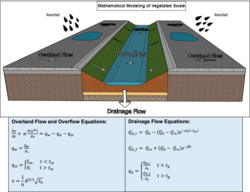

## INTRODUCTION

An increase in extreme weather conditions because of climate change affects a large part of the world and results in severe flooding events, particularly in urbanized areas involving heavily human‐driven effects such as buildings, roads, and parking lots (Intergovernmental Panel on Climate Change (IPCC), 2023). Changing rainfall characteristics under the influence of climate change can cause stormwater drainage systems to exceed their capacities, and therefore, aging infrastructure systems are incapable of sustainable stormwater management (Duin et al., 2021). Consequently, there is a growing need for new measures of stormwater runoff control. Green infrastructures, also known as Low Impact Development (LID), are eco‐friendly systems that can decrease surface runoff and control water quality (Godyń et al., 2024; Yang and Li, 2013). Many studies have been conducted in the last two decades, including fieldwork and numerical modeling of LID (Cao et al., 2023). However, there is still room for improvement in the hydrological modeling of different types of LID including swales.

Vegetated swales, also known as vegetated open channels, are one of the most implemented types of LID, which can be used as a natural instrument to convey, infiltrate, and store surface runoff. It is crucial to accurately understand the physical behavior of vegetated swales and model swale hydrology for efficient design and implementation. For this reason, several software packages incorporated modules specifically designed for LID simulations, such as EPA SWMM, PCSWMM, SUSTAIN, MUSIC, MIKE SHE, HEC‐HMS, SWAT, and WinDes® (Agarwal et al., 2024; Fowdar et al. 2022; Gao et al., 2015; Khaniya et al., 2017; Kokas, 2017; Lashford et al., 2014; Rujner et al., 2018; Seo et al., 2017; Shannak, 2021; Xie et al., 2017). Thus, these tools can be employed to simulate vegetated swales.

Among the aforementioned models, EPA SWMM, HEC‐HMS, and SWAT stand out as the most widely used because of their accessibility as free downloads. However, the vegetated swale in EPA SWMM is characterized by a limited set of parameters, lacking specific inputs for defining soil, storage, and drainage properties. SWAT incorporates LIDs such as filter strips, grassed waterways, and infiltration trenches into the model by assigning specific land cover types to Hydrologic Response Units (HRUs) and analyzes water quantity, quality, and sediment transport. Similar to SWAT, HEC‐HMS utilizes Curve Numbers (CN) to represent the hydrological behavior of LIDs within subbasins. In other words, there are no separate modules particularly designed for modeling LIDs in both SWAT and HEC‐HMS, that is, they integrate LID effects within their broader hydrological modeling framework. This approach brings several disadvantages, such as low resolution and lack of flexibility in changing swale properties. Besides EPA SWMM, HEC‐HMS, and SWAT, MUSIC and MIKE SHE are also frequently used in LID simulation. The MUSIC model focuses on storage–discharge relationships using Manning's equation. MIKE SHE by DHI is also capable of modeling swale hydrology. The disadvantages of MIKE SHE are high‐resolution requirements and complicated calibration and validation processes. Eventually, each model has both advantages and disadvantages and thus needs improvement for accurate simulations of the hydrological behavior of vegetated swales.

Besides software packages, there are several individual models that have been developed to specifically analyze LID hydrology. Grinden (2014) developed a numerical model for swales using MATLAB in which overland flow is solved by St. Venant's equations and infiltration on side slopes is described by Green–Ampt equations. In this model, the inflow hydrograph entering the swale was defined as an input parameter rather than being calculated as overland flow generated over a watershed surface. In addition, model's capability and accuracy require validation through the application of experimental data. Chen et al. (2017) developed an integrated model of overland flow, infiltration, and underdrain for vegetated dry swale hydraulics, which takes both gravel and perforated pipe systems into account. In this model, infiltration was calculated with the 1‐D Richards equation, but the horizontal flow and drainage in the soil layer were neglected. The study by Chijioke Ekwu (2021) presented the development of a simple model with few parameters for Blue–Green Systems, which adopts the Nonlinear Reservoir Model to determine the hydraulic behavior of Blue–Green Systems. Table 1 shows a summary of existing models and their gaps in literature.

**TABLE 1:** Summary of literature review.

Reference	Research focus	Methods used	Identified gaps
Deletic (2001)	Surface runoff and transport dynamics in vegetated areas	Empirical and simulation modeling	Lack of comprehensive testing across different grass types; limited sediment analysis
Deletic and Fletcher (2006)	Field performance of grass filters for sediment removal	Empirical modeling (TRAVA)	Focuses on the performance of grass filters
Wong et al. (2006)	Unified modeling for various stormwater treatment systems	CSTR‐based modeling	Requires additional calibration for different treatment systems; limited adaptability to varying climate zones
Abida et al. (2007)	Analysis of grass swales integrated with perforated pipe systems	Hydraulic modeling	Inadequate assessment of different rainfall scenarios; limited field data for calibration
Ackerman and Stein (2008)	Effectiveness of BMPs in stormwater management	Dynamic modeling over 10‐year period	Limited model sensitivity analysis; lacks data on BMP performance during extreme events
Lashford et al. (2014)	Sustainable drainage management strategies	WinDes®	Absence of detailed field experiments; limited regional adaptation discussions
Grinden (2014)	Combined hydraulic and infiltration modeling for swales	Explicit discretization models	Computational instabilities; absence of data for model validation
Gao et al. (2015)	Application of best management practices (BMPs) for urban runoff control	SUSTAIN	Not include calibration of the flow model, it needed monitoring data to conduct detailed calibration process
Chen et al. (2017)	Simulation of hydraulic performance in underdrain systems	Combined flow and infiltration modeling	Simplified assumptions for underdrain behavior; insufficient validation with diverse soil types
Khaniya et al. (2017)	Evaluate LID and BMP design performance in stormwater management.	HEC‐HMS	Defining LID and BMP by using curve number at the sub‐catchment scale
Seo et al. (2017)	Evaluate the impact of different LID practices	SWAT	Defining LID with specific landcover types at the sub‐catchment scale
Xie et al. (2017)	Combined use of swales and permeable pavements	SWMM	Absence of long‐term data for varying rainfall intensities; limited integration with urban planning frameworks
Kokas (2017)	Impact of land use and LID measures on flood hazards	SWMM	Narrow scope of data analysis; absence of consideration for climate change impacts on future scenarios
Rujner et al. (2018)	Hydrological response of grass swales using high‐resolution models	Mike SHE	High computational demand; challenges in scaling up results for larger systems
Chijioke Ekwu (2021)	Simulation of blue–green stormwater infrastructure	Dynamic modeling	Limited validation data; lacks consideration of long‐term climate variations and different urban settings
Shannak (2021)	Investigate LID practices' role in urban aquatic ecosystem management, specifically their effect on aquatic life in Blunn Creek, Texas.	SWAT	Defining LID with specific landcover types at the sub‐catchment scale
Fowdar et al. (2022)	Evaluation of green infrastructure for stormwater management	MUSIC	Insufficient model transferability; underestimation of sediment load variability
Beceiro et al. (2022)	Evaluating the role of NBS in enhancing urban resilience	Mathematical modeling	Lack of comparative analysis with other NBS; insufficient long‐term performance data
Agarwal et al. (2024)	Assess the effectiveness of nature‐based solutions (NbS) for peak flow reduction in river catchments	HEC‐HMS	Defining nature‐based solutions by using curve number at the sub‐catchment scale

In addition to these modeling efforts, integrated water quantity and quality modeling of swales have also been practiced. Deletic (2001) developed a mathematical model that involves simulations of surface runoff and sediment transport over grass surfaces under non‐submerged flow conditions. Then, Deletic and Fletcher (2006) developed a model called TRAVA to determine the total suspended solids treatment performance of grass filters, which have different geometry than grass swales. Wong et al. (2006) developed an algorithm for flow hydrodynamic and water‐quality analyses that can reveal the treatment performance of different LID by employing a first‐order kinematic decay model. Abida et al. (2007) developed a computer model called ANSWAPPS, which aims to analyze and design an integrated grass swale‐perforated pipe system. Ackerman and Stein (2008) developed a dynamic model to determine the volumetric reduction capacity, sediment, and total copper treatment performance of retention and swale.

To contribute to the existing research efforts on swales, this study aims to develop a mathematical model that can simulate vegetated swale hydrology by determining overland flow, overflow, and drainage flow. The hydrological model consists of two main modules: the Swale Overflow Model and the Swale Drainage Model. The Drainage Model represents the rising and falling limbs of the drainage flow with different forms of exponential functions, and calculated drainage flow values feed the Swale Overflow Model. The Swale Overflow Model engages the Kinematic Wave Method to simulate the overflow by taking into account the overland flow reaching the swale from the watershed as a water source, the drainage flow at the bottom of the swale, and the swale storage as a water sink. Finally, data sets from the Rainfall–Watershed–Swale (RWS) experimental system were employed in developing and calibrating the hydrological model.

## MATERIALS AND METHODS

### Rainfall‐Watershed‐Swale experimental system

The RWS experimental system was constructed at the Istanbul University – Cerrahpaşa Campus to observe and understand the hydrological behavior of vegetated swales. Experimental data obtained from the RWS was made use in developing the hydrological model for vegetated swales. The RWS is a large‐scale outdoor experimental setup and consists of three different components, that is, a sprinkler system, a watershed area, and a swale channel. The sprinkler system enables the simulations of different types of rainfall events with different intensities and areal distributions on the watershed. The overland flow generated over the watershed, which has a 0.7% surface slope and 40‐m^2^ surface area, is transmitted to the vegetated swale. Part of the overland flow is turned into the overflow over the swale surface while the rest of the water infiltrates, resulting in storage in the swale's soil body, and percolates through the swale layers, resulting in drainage flow. In order to investigate the effects of different parameters on swale hydrology, several experiments were conducted with rainfall intensities of 20 and 30 mm/h; rainfall durations of 15 and 25 min; grass heights of 7 and 11 cm; and areal rainfall scenarios of R‐A, R‐B, R‐C, and R‐D, and overflow and drainage flow were measured. The swale module of the RWS consists of several layers, which are the gravel layer (10 cm), sand layer (5 cm), vegetative soil‐coarse sand mixture layer (45 cm), and grass surface layer, respectively. The rainfall intensity was selected within the limits allowed by the RWS experimental system, and two selected intensities represent moderate and extreme storm events. The rainfall duration was chosen by considering the time of concentration. The grass height was selected considering the climatic conditions of the region where the experiments were conducted. A summary of the design parameters is presented in Table 2.

**TABLE 2:** Design parameters.

Design parameter	Value
Rainfall intensity, *i* (mm/h)	20 and 30
Areal rainfall scenarios	R‐A, R‐B, R‐C, and R‐D
Rainfall duration (min)	15 and 25
Width of the watershed (m)	4
Length of the watershed (m)	10
Surface slope of watershed, S_0_ (m/m)	0.0007
Top width of swale (mm)	140
Bottom width of swale (mm)	50
Surface slope of swale, S (%)	5
Side slope of swale (m/m)	1:3

**FIGURE 1:**
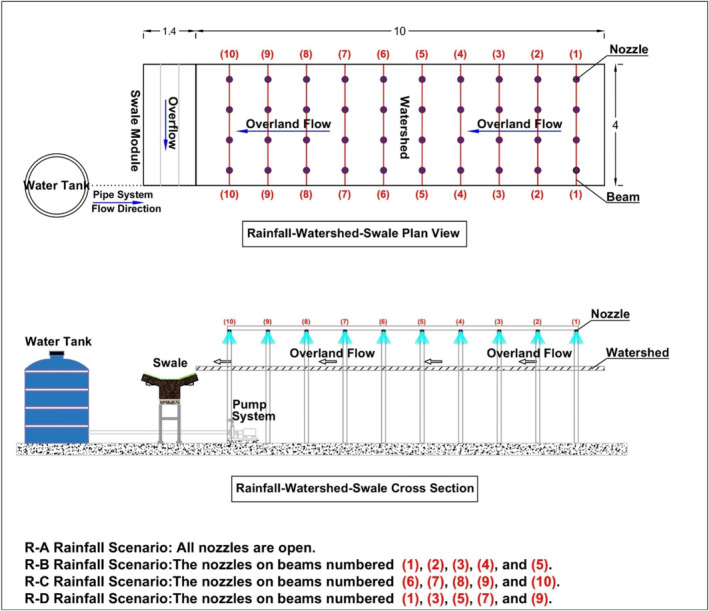
Schematic view of the Rainfall–Watershed–Swale experimental system (modified after Saraçoğlu & Kazezyılmaz‐Alhan, 2023).

**FIGURE 2:**
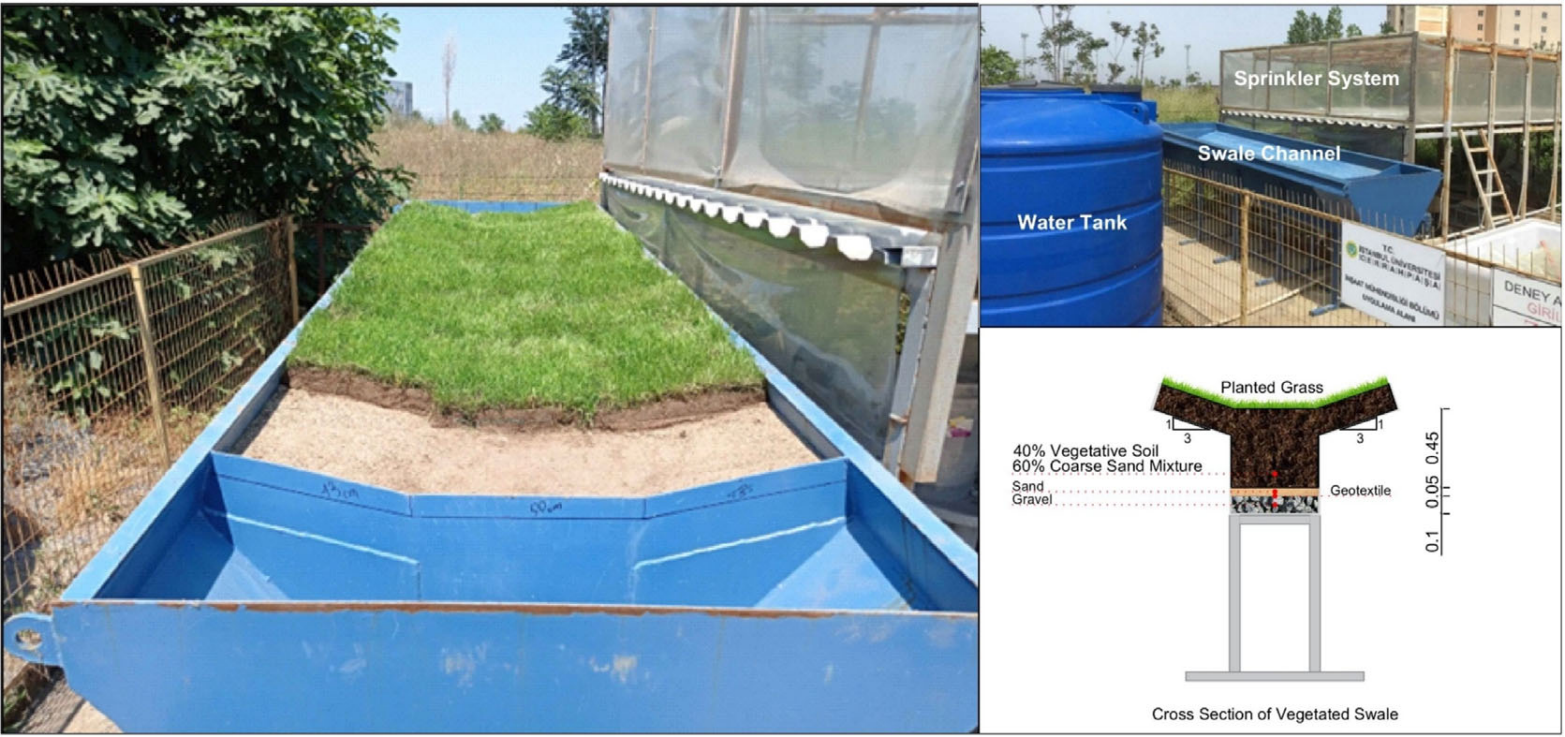
Rainfall–Watershed–Swale experimental system (modified after Saraçoğlu & Kazezyılmaz‐Alhan, 2023).

The schematic plan and cross‐section of the RWS, vegetated swale, and rainfall scenarios are presented in Figure 1. Figure 2 shows the RWS experimental system. Detailed information regarding the RWS and test results can be found in Saraçoğlu and Kazezyılmaz‐Alhan (2023).

### Development of a hydrological model

**FIGURE 3:**
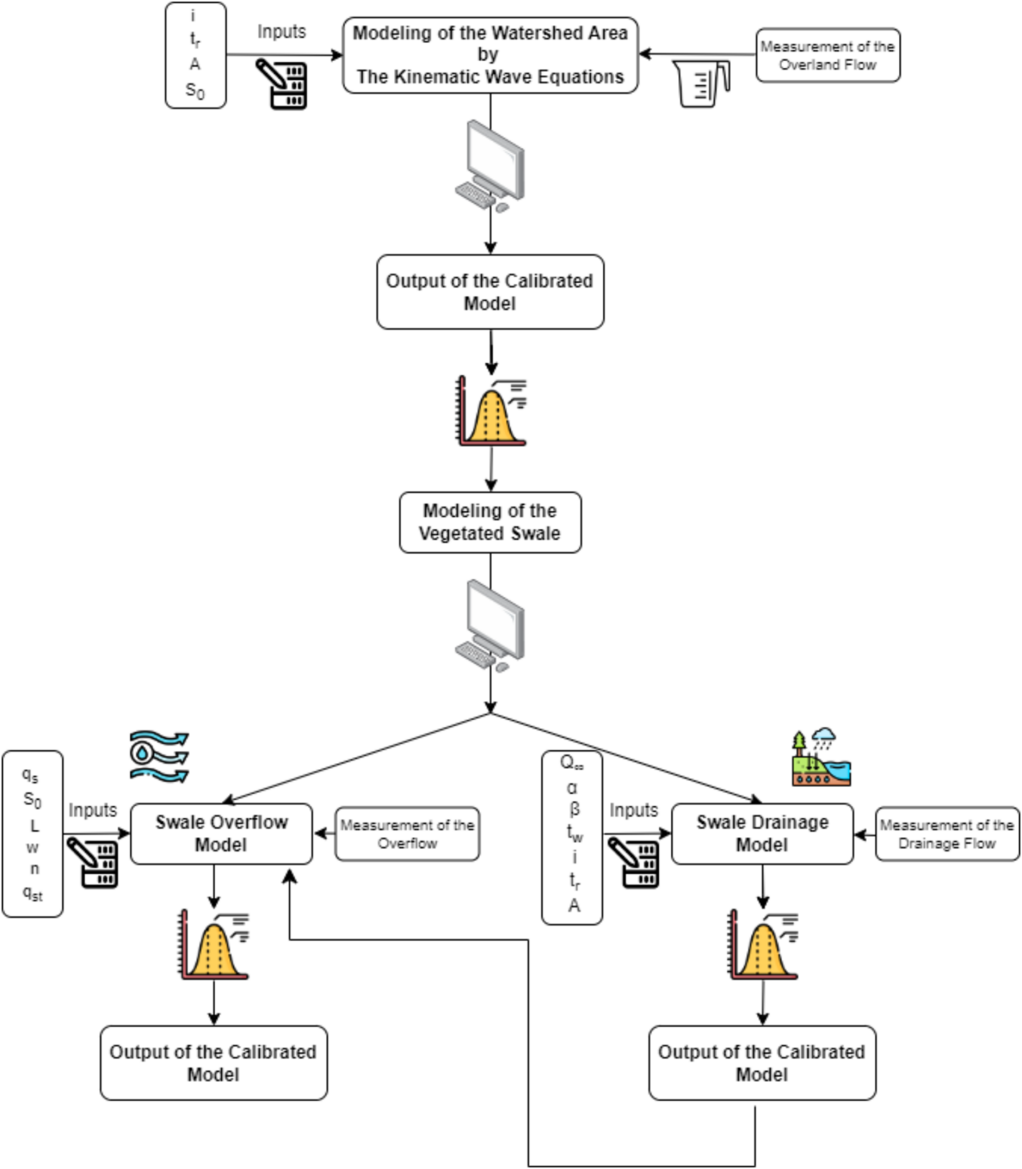
Modeling steps of the watershed area and the vegetated swale.

Vegetated swales control overland flow and surface runoff by storing and draining the receiving water and conveying the rest as overflow over the swale surface. Therefore, the equations used for modeling of swale hydrology should represent all these components. Consequently, the developed hydrological model consists of two coupled modules: (1) Swale Overflow Model and (2) Swale Drainage Model. The Swale Overflow Model simulates the flow generated over the swale surface, while the Swale Drainage Model calculates the amount of water drained under the swale. The flow chart summarizing all steps of the hydrological model of the vegetated swale, including modeling of the watershed area, is presented in Figure 3.

The Swale Overflow Model calculates the overflow rate by employing the kinematic wave equation and Manning's equation. The overland flow generated over the watershed, the stored water in the swale, and the drained water through the swale are taken into account by incorporating the overland flow rate as a water source and the drainage and storage rate as a water sink in the kinematic wave equation. The governing equations for the Swale Overflow Model are as follows:

(1) $$\left.\left(\begin{array}{c} \frac{\partial y}{\partial t}+\frac{\partial q_{s}}{\partial x}=q_{w}-q_{d}-q_{\mathrm{st}}\\ \;q_{s}=\alpha \;y^{m} \end{array}\right)\right\}\;\frac{\partial y}{\partial t}+\alpha \frac{\partial \left(y^{m}\right)}{\partial x}=q_{w}-q_{d}-q_{\mathrm{st}}$$

(2) $$q_{s}=\alpha \;y^{m}$$

(3) $$q_{\mathrm{st}}=\left\{\begin{array}{c} S_{\mathrm{st}},\;t<t_{\mathrm{st}}\\ 0,\;t>t_{\mathrm{st}} \end{array}\right.$$

(4) $$v=\frac{1}{n}R^{2/3}\sqrt{S_{0}}$$

(5) $$\alpha =\frac{\sqrt{S_{o}}}{n}\;\text{and}\;m=5/3$$

where y is the water depth (*L*) over the swale, $q_{s}$ is the flow rate per unit width over the swale surface ($L^{2}/T$), t is the time (T), x is the length (L), $q_{w}$ is the overland flow rate over the watershed receiving unit swale area ($L^{3}/L^{2}T)$, $q_{d}$ is the drainage rate ($L^{3}/L^{2}T$), $\;q_{\mathrm{st}}$ is the storage rate $(L^{3}/L^{2}T$), $S_{\mathrm{st}}$ is the storage capacity of the swale ($L^{3}/L^{2}T$), $t_{\mathrm{st}}$ is the residence time of the swale storage $\left(T\right),$ $A_{s}$ is the swale surface drainage area ($L^{2}$), v is the flow velocity over the swale surface (L/T), n is the Manning's coefficient for the vegetated swale surface, Ris the hydraulic radius (*L*), and α and m are the coefficients.

By using the Swale Overflow Model, the overflow rate at the outlet of the vegetated swale is calculated. The overland flow is first calculated and used as an input in the Swale Overflow Model. The drainage rate is also calculated using the Swale Drainage Model, and the output is used as input in the Swale Overflow Model as well. The solution of the Swale Overflow Model was accomplished by using the implicit finite difference method in MATLAB. The application of the implicit finite difference method to the equations of the Swale Overflow Model is presented as follows:

(6) $$\begin{array}{c} \frac{{q_{s}}_{i+1}^{j+1}-{q_{s}}_{i}^{j+1}}{∆x}+\frac{y_{i+1}^{\;j+1}-y_{i+1}^{\;j}}{∆t}={\left(q_{w}-q_{d}-q_{\mathrm{st}}\right)}_{i+1}^{\;j}\\ {q_{s}}_{i+1}^{j+1}=\alpha {\left(y_{i+1}^{\;j+1}\right)}^{m}\\ {q_{s}}_{i}^{j+1}=\alpha {\left(y_{i}^{\;j+1}\right)}^{m} \end{array}$$

(7) $$f\;\left(y_{i+1}^{\;j+1}\right)=\frac{∆t}{∆x}\alpha {\left(y_{i+1}^{\;j+1}\right)}^{m}+y_{i+1}^{\;j+1}-\left[\frac{∆t}{∆x}\alpha {\left(y_{i}^{\;j+1}\right)}^{m}+y_{i+1}^{\;j}+∆t{\left(q_{w}-q_{d}-q_{\mathrm{st}}\right)}_{i+1}^{\;j+1}\right]$$

The unknown water depth over the swale surface, $y_{i+1}^{\;j+1}$, is solved in the Swale Overflow Model by the Newton–Raphson iteration method. The iteration steps regarding the Newton–Raphson method are given in the following equations:

(8) $${\left(y_{i+1}^{\;j+1}\right)}_{k+1}={\left(y_{i+1}^{\;j+1}\right)}_{k}-\frac{f{\left(y_{i+1}^{\;j+1}\right)}_{k}}{f^{\prime }{\left(y_{i+1}^{\;j+1}\right)}_{k}}\;\left|f{\left(y_{i+1}^{\;j+1}\right)}_{k+1}\right|\leq \varepsilon$$

(9) $$f^{\prime }\left(y_{i+1}^{\;j+1}\right)=1+\mathrm{\alpha m}\frac{∆t}{∆x}\;{\left(y_{i+1}^{\;j+1}\right)}^{m-1}$$

The Swale Drainage Model was developed to calculate the drainage flow rate at the outlet of the swale bottom. In order to develop the Swale Drainage Model, the drainage characteristics of the swale were examined by employing drainage measurements obtained from the experiments. The measured data show that the drainage behavior of the vegetated swale may be represented in two parts. Thus, two different exponential functions, which represent the rising limb and falling limb patterns of drainage flow separately, are considered for best fitting.

The exponential functions representing the rising limb and falling limb of the drainage hydrograph of the vegetated swale are given in Equations (10)–(12), respectively.

(10) $$Q_{d\_r}=Q_{0}-\left(Q_{0}-Q_{\infty }\right)e^{-\alpha \left(t-t_{w}\right)}$$

(11) $$Q_{d\_f}=Q_{\infty }+\left(Q_{0}-Q_{\infty }\right)e^{-\mathrm{\beta t}}$$

(12) $$q_{d}=\left\{\begin{array}{c} \frac{Q_{d\_r}}{A_{s}}\;t<t_{p}\\ \frac{Q_{d\_f}}{A_{s}}\;t>t_{p} \end{array}\right.$$

In Equations (10)–(12), $Q_{d\_r}$ is the drainage flow for the rising limb part ($L^{3}/T),Q_{d\_f}$ is the drainage flow for the falling limb part ($L^{3}/T),$ $Q_{0}$ is the maximum drainage flow rate $\left(L^{3}/T\right)$, $Q_{\infty }$ is the minimum drainage flow rate ($L^{3}/T)$, α is the exponent of the rising limb (1/T), β is the exponent of the falling limb (1/T), *t* is the duration (T), $t_{w}$ is the time that the drainage flow starts at the outlet of the swale bottom (T), and *t*_*p*_ is the time that drainage flow rate reaches its maximum value.

The terms $Q_{d\_r}$ and $Q_{d\_f}$, which represent the drainage flow rates for the rising limb and falling limb of the drainage hydrograph, are functions of time, and the exponents of α and β, where α and β reflect vegetated swale characteristics and take different values for different types of swales. The exponent *α* represents the ascending drainage capacity of the vegetated swale, whereas the exponent *β* represents the descending drainage capacity of the vegetated swale. In addition, the exponents *α* and *β* describe both the properties of the soil and surface layers. The exponential functions of $e^{-\alpha \left(t-t_{w}\right)}$ and $e^{-\mathrm{\beta t}}$ determine the rise and fall characteristics of the drainage flow, respectively. The term $t_{w}$ depends on the swale type and gives the time when the drainage flow begins at the outlet of the drainage flow.

## RESULTS

The hydrological model for swale presented in the previous section is applied for the cases of the experiments conducted in the RWS experimental setup (Saraçoğlu & Kazezyılmaz‐Alhan, 2023) for different rainfall conditions, and numerical results are compared with experimental data to present the capability and accuracy of the developed model. The input data for the Swale Drainage Model include $Q_{0}$, $Q_{\infty }$, α, β, *t*_*p*_ and $t_{w},$ and the input data for the Swale Overflow Model include $q_{d}$, $q_{s},q_{\mathrm{st}}$ and *n*. These parameters depend primarily on the swale type and therefore should be determined based on the experimental results. In addition, minimum and maximum drainage flow rates may also depend on the rainfall type. Hence, we determined the values of these parameters step by step.

First, for the Swale Drainage Model, by using the measured minimum and maximum drainage flow rates, the optimum values for α, β, and $t_{w}$ were calculated using MATLAB for each experiment. Optimization was applied by utilizing the “lsqcurvefit” function to minimize the differences between the measured drainage flow rates during experiments and the drainage flow rates calculated by the Swale Drainage Model. Table 3 presents the measured minimum and maximum drainage flow rates in the experiments and different rainfall conditions for each experiment, such as rainfall intensities (20 and 30 mm/h), rainfall durations (15 and 25 min), and areal rainfall scenarios (R‐A, R‐B, R‐C, and R‐D).

**TABLE 3:** Experimental sets, minimum and maximum drainage flow rates, and coefficients.

Experiment no.	Rainfall intensity (mm/h)	Rainfall duration (min)	Rainfall scenario	$Q_{0}$ (1/min)	$Q_{\infty }$ (1/min)	α (1/min)	β (1/min)	$t_{w}$ (min)
1	20	25	R‐A	0.756	0.068	0.1747	0.0153	14.0233
2	20	15	R‐A	0.540	0.047	0.1869	0.012	13.5085
3	20	25	R‐A	0.720	0.073	0.1601	0.0158	13.9093
4	20	25	R‐B	0.620	0.069	0.2907	0.0159	15.0719
5	20	25	R‐C	0.610	0.051	0.1136	0.0124	14.1171
6	20	25	R‐D	0.620	0.070	0.1052	0.0142	14.8140
7	20	15	R‐D	0.403	0.080	0.0927	0.012	15.0809
8	30	25	R‐A	0.806	0.066	0.1616	0.0157	12.5318
9	30	15	R‐A	0.560	0.048	0.1464	0.0121	11.1271
10	30	25	R‐A	0.757	0.080	0.1382	0.018	12.6501
11	30	25	R‐B	0.620	0.070	0.2906	0.016	15.0751
12	30	25	R‐C	0.636	0.060	0.1093	0.012	14.3351
13	30	25	R‐D	0.645	0.076	0.0999	0.0165	14.7966
14	30	15	R‐D	0.420	0.078	0.0913	0.012	15.2747

During the experiments, drainage flow rates were measured every 15 min for 6 h at the outlet of the swale bottom. Drainage flow rates measured at the end of the sixth hour represent the minimum drainage flow rates in the model. There are no significant differences among the minimum drainage flow rates for different experiments (See Table 3). Therefore, the value of the minimum drainage flow rate $\left(Q_{\infty }\right)$ is taken as the average of all the minimum drainage flow rates measured during the experiments. Regarding the maximum flow rates, we observe that the peak drainage flow rates vary with respect to the rainfall intensities, rainfall durations, and areal rainfall scenarios. Areal rainfall scenarios, in fact, reflect the surface area of the watershed where rainfall directly drops. The relationship of the peak drainage flow rate was obtained with an empirical equation as a function of rainfall intensity, rainfall duration, and the effective watershed area by using multiple regression analysis as follows:

(13) $$Q_{0}=0.0165\;x\;i^{0.0964}\;x\;t_{r}^{0.7306}\;x\;A^{0.3213}\;$$

where $Q_{0}$ is the maximum drainage flow rate ($L^{3}/T)$, i is the rainfall intensity (L/T), $t_{r}$ is the rainfall duration (T), and A is the effective watershed area ($L^{2}$).

Next, for the Swale Overflow Model, by using the measured overflow hydrograph and calculated drainage flow, the optimum values for Manning's coefficient $\left(n\right)$ and the storage rate $\left(q_{\mathrm{st}}\right)$ are obtained with manual calibration. During the calibration, the minimum difference between the model outputs and the measurements was satisfied. The calibrated values for the parameters of Manning's coefficient $\left(n\right)$, the storage capacity $\left(S_{\mathrm{st}}\right)$, the minimum drainage flow rate ($Q_{\infty })$, the exponent of the rising limb (*α*), the exponent of the falling limb (*β*), and the time that the drainage flow starts at the outlet of the swale bottom ($t_{w}$) are presented in Table 4.

**TABLE 4:** Calibrated parameters of the swale hydrological model.

Parameters	Values
*n*	0.4
$S_{\mathrm{st}}$ (cm/min)	2
$Q_{\infty }$(l/min)	0.067
α (1/min)	0.15
β (1/min)	0.014
$t_{w}$ (min)	14

**FIGURE 4:**
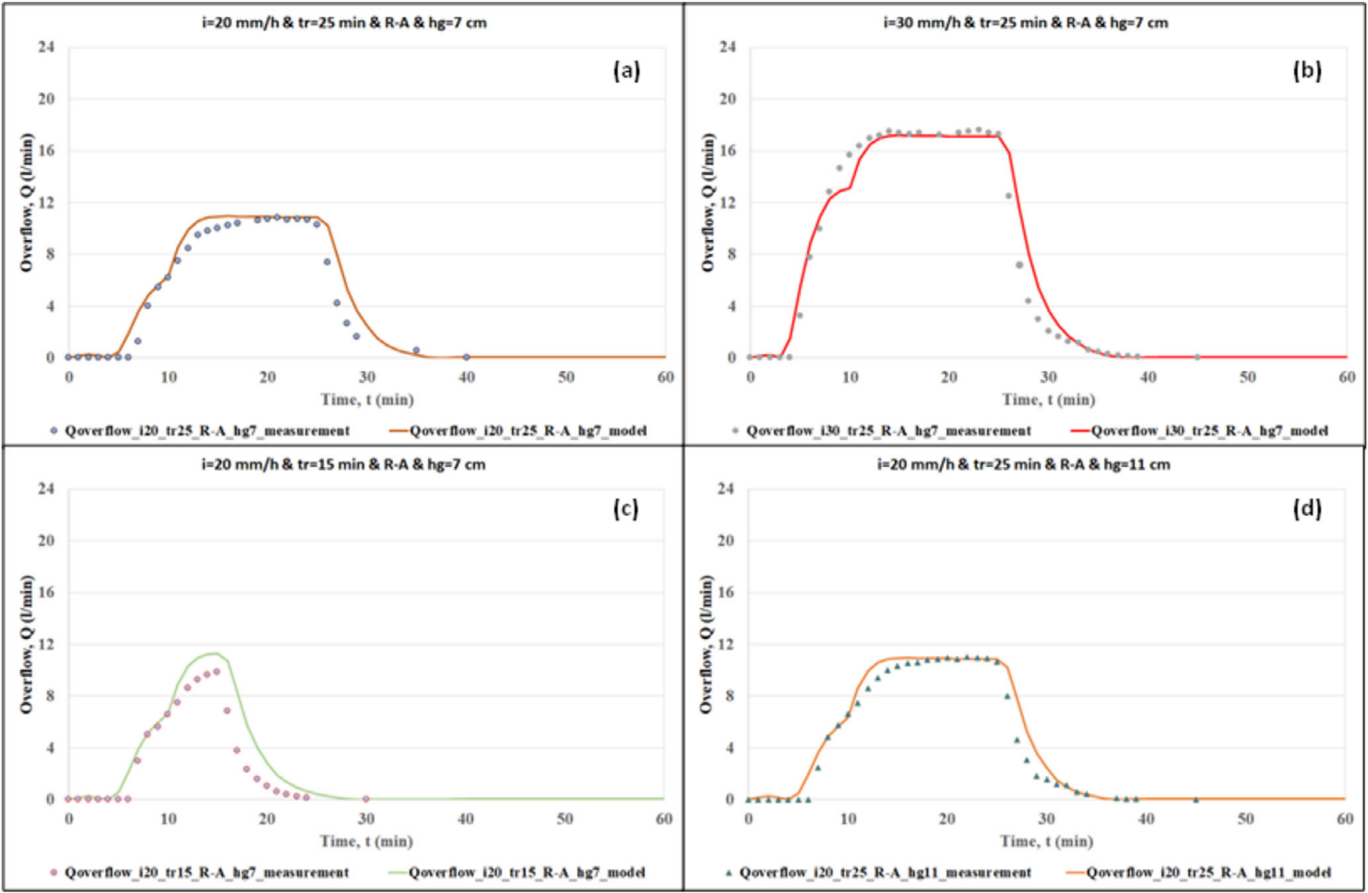
Comparisons of model outputs with overflow data (*i* = 20 and 30 mm/h, *tr* = 15 and 25 min, *hg* = 7 and 11 cm).

**FIGURE 5:**
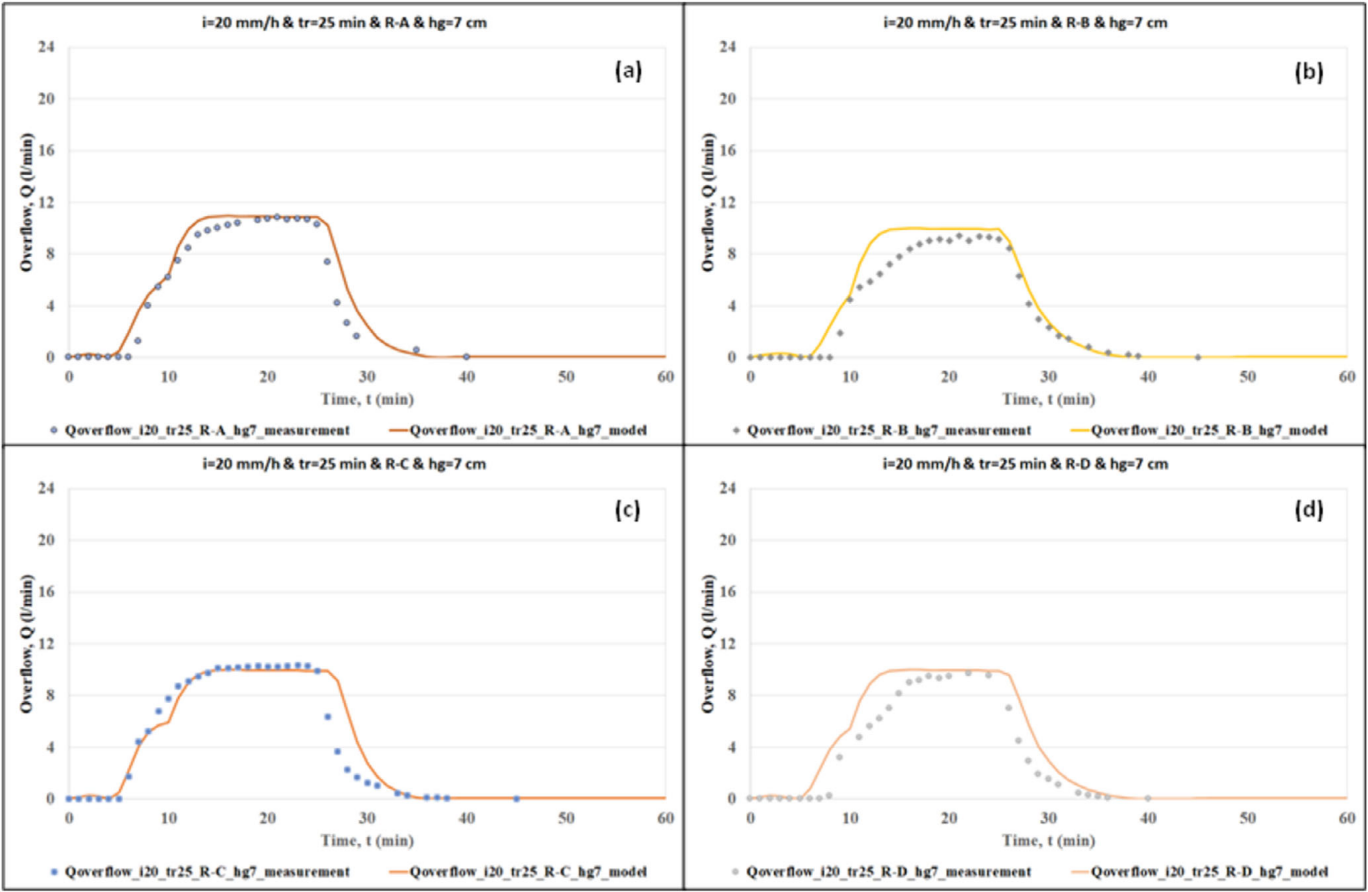
Comparisons of model outputs with overflow data (*i* = 20 mm/h, *tr* = 25 min, *hg* = 7 cm, and R‐A, R‐B, R‐C, R‐D).

**FIGURE 6:**
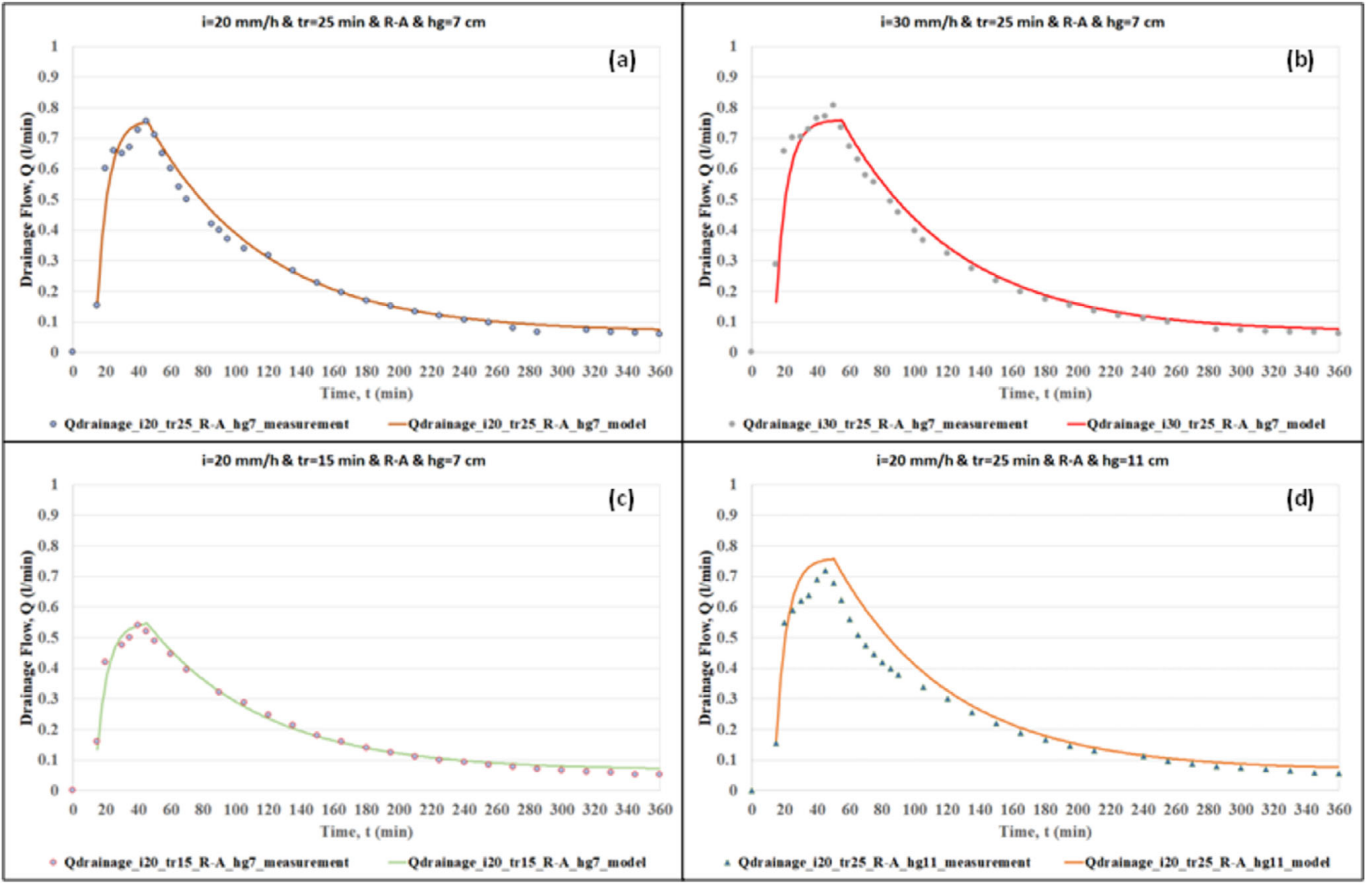
Comparisons of model outputs with drainage flow data (*i* = 20 and 30 mm/h, *tr* = 15 and 25 min, *hg* = 7 and 11 cm).

**FIGURE 7:**
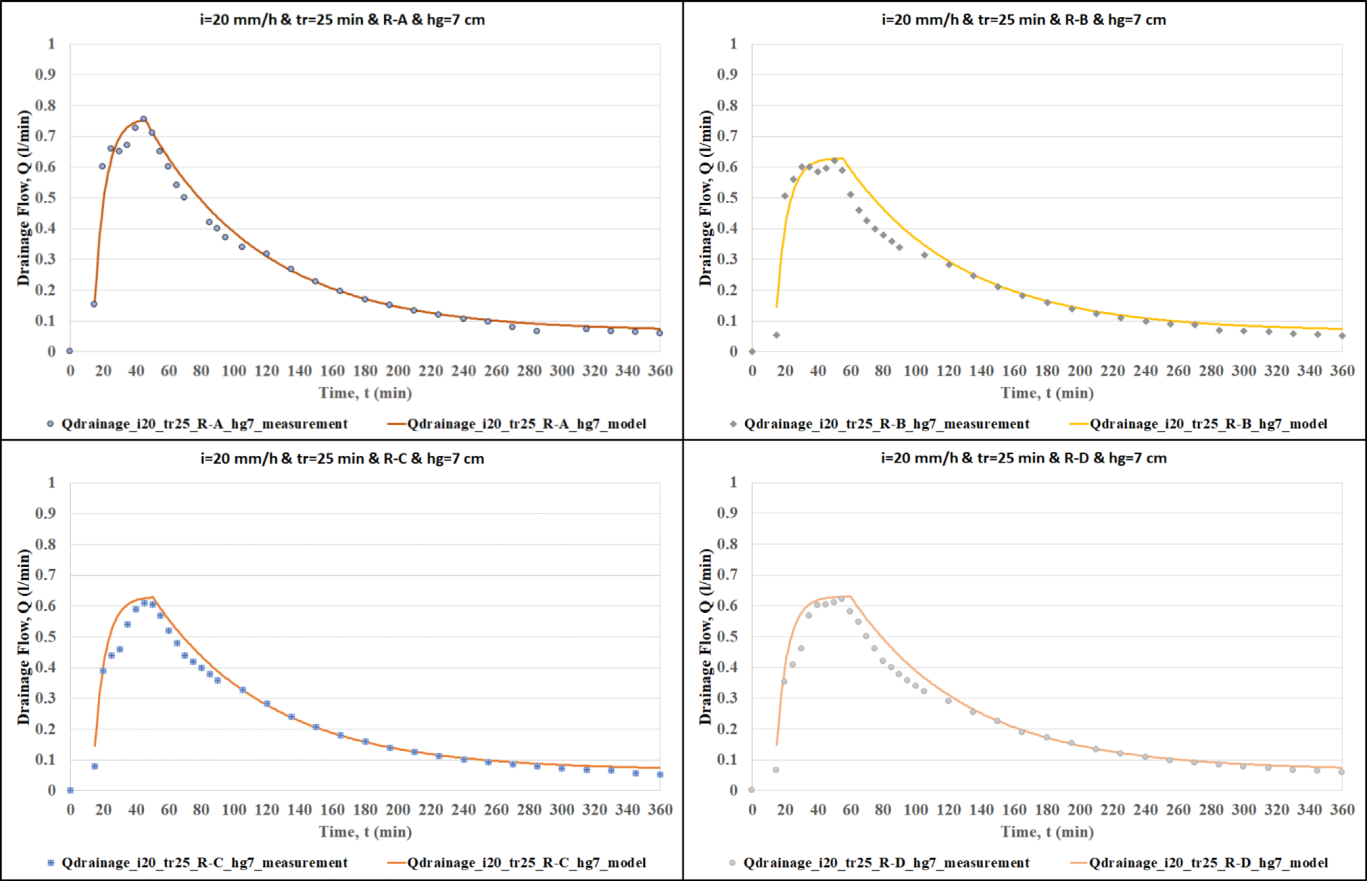
Comparisons of model outputs with drainage flow data (*i* = 20 mm/h, *tr* = 25 min, *hg* = 7 cm, and R‐A, R‐B, R‐C, R‐D).

The effectiveness of the hydrological model was demonstrated by comparing the outputs of the model with the measurements of the RWS experiments. The comparison of the model outputs and the measurements of overflow and drainage flow are given in Figures 4, 5, 6, 7.

Figures 4 and 5 show the comparison of calculated overflow rates with the measured data for different rainfall and swale conditions. Figure 4 shows the overflow hydrograph for the cases of rainfall intensities of 20 and 30 mm/h, rainfall durations of 15 and 25 min, and grass heights of 7 and 11 cm under areal rainfall scenario R‐A. The comparison of Figure 4a with Figure 4b shows the role of rainfall intensity in modeling accuracy. As the rainfall intensity increases, the difference between the calculated hydrograph and the measured hydrograph slightly decreases. The comparison of Figure 4a with Figure 4c shows the role of rainfall duration in modeling accuracy. As the rainfall duration increases, the difference between the calculated hydrograph and the measured hydrograph decreases. In addition, the calculated peak overflow rate catches the measured one in a similar way. Consequently, in case of extreme rainfall events, one can expect slight deviations between the data and the model results. The comparison of Figure 4a with Figure 4d shows the role of grass height in modeling accuracy. During the experiments done by Saraçoğlu and Kazezyılmaz‐Alhan (2023), the overflow depth developed over the swale surface was smaller than the grass height, which resulted in negligible differences in the experimental results. In other words, the effect of grass height could not be observed. Consequently, the calculated results are also very similar for the cases with different grass heights.

The effect of different areal rainfall scenarios (R‐A, R‐B, R‐C, and R‐D) on the model outputs can be revealed by examining the graphs in Figure 5. The model simulated the peak overflow rates accurately under the areal rainfall scenarios of R‐A, R‐C, and R‐D. Moreover, under R‐A and R‐C, the rising limb of the hydrograph is determined quite well compared to the rising limbs of other scenarios. The calculated peak overflow rate under R‐B is slightly higher than the measured peak overflow rate.

Figures 6 and 7 show the performance of the hydrological model in drainage flow calculation. These graphs show that the Swale Drainage Model effectively simulates the drainage flow in the swale. Based on the comparison of Figure 6a with Figure 6b, one can claim that the change in rainfall intensity did not cause a significant difference in the pattern of drainage hydrographs, and in both graphs, there is a very good agreement between the measured and calculated drainage flow rate. Comparison of Figure 6a with Figure 6c shows that even though the change in rainfall duration affects the peak drainage flow, it does not have an impact on the modeling performance, that is, there is a very good agreement between the measured and calculated drainage flow rate for both cases. Finally, based on the comparison of Figure 6a with Figure 6d, a slight difference is observed between measured and calculated drainage flow values for large grass height.

The effects of different areal rainfall scenarios on the drainage flow calculation are shown in Figure 7. The best hydrological model performance is demonstrated for the rainfall scenario of R‐A. Yet, there are still good matches between the measured and calculated values for the rest of the rainfall scenarios.

The goodness of fit measures, that is, the Determination Coefficient (*R* ^2^), Root Mean Square Error (RMSE), and Nash–Sutcliffe Efficiency (NSE), were calculated. The goodness of measures represents the differences between the hydrological model outputs and measurements. Table 5 shows the *R* ^2^, RMSE, and NSE values for both overflow and drainage flow hydrographs. The results in Table 5 indicate that the Swale Model can accurately simulate both the overflow and drainage flow behavior of the vegetated swale.

**TABLE 5:** Statistical analysis of the hydrological model outputs of overflow and drainage flow.

Experiment no.	Rainfall intensity (mm/h)	Rainfall duration (min)	Rainfall scenario	Grass height (cm)	Overflow			Drainage flow		
					*R* ^2^	RMSE	NSE	*R* ^2^	RMSE	NSE
1	20	25	R‐A	7	0.954	1.282	0.917	0.985	0.033	0.982
2	20	15	R‐A	7	0.922	1.725	0.764	0.988	0.020	0.987
3	20	25	R‐A	11	0.970	0.954	0.957	0.981	0.060	0.931
4	20	25	R‐B	7	0.964	1.222	0.897	0.963	0.048	0.947
5	20	25	R‐C	7	0.898	1.452	0.891	0.988	0.036	0.966
6	20	25	R‐D	7	0.921	1.756	0.787	0.983	0.044	0.950
7	20	15	R‐D	7	0.965	1.952	0.418	0.930	0.034	0.920
8	30	25	R‐A	7	0.969	1.390	0.966	0.970	0.046	0.970
9	30	15	R‐A	7	0.928	1.870	0.920	0.975	0.030	0.974
10	30	25	R‐A	11	0.971	1.307	0.971	0.964	0.063	0.924
11	30	25	R‐B	7	0.975	1.024	0.974	0.963	0.048	0.947
12	30	25	R‐C	7	0.902	2.018	0.900	0.982	0.028	0.981
13	30	25	R‐D	7	0.949	1.524	0.937	0.982	0.041	0.957
14	30	15	R‐D	7	0.921	1.722	0.887	0.940	0.033	0.935

## DISCUSSION

This study aims to develop a hydrological model that can simulate the overflow and drainage flow of vegetated swale. The developed model consists of the “Swale Overflow Model” and the “Swale Drainage Model,” which are coupled modules, and a code for these two modules was written in MATLAB. Several hydrological parameters were considered in the development of the model. When the effects of different areal rainfall scenarios are evaluated on the Swale Overflow Model, there is quite good agreement between the model outputs and measurements for scenarios R‐A and R‐C. However, under the R‐B and R‐D scenarios, slight differences between the overflow rates and the measured data, particularly in the rising limb and falling limb parts of the hydrographs, are observed. Rainfall intensity and duration are also effective on swale hydrology. In addition, it is observed that the storage capacity of swale has also a significant effect on swale overflow.

With regard to the vegetation condition of the swale, the model takes the effect of vegetation into account only with Manning's coefficient. However, there may be other potential parameters that are effective in swale hydrology. For example, the grass height is effective not only on the flow over the swale surface but also the existence of different roots for different grass heights should also be effective on drainage flow. Further study should investigate the said effects and include an additional variable in the developed model to represent this situation. During the calibration of the overflow model, it was observed that Manning's coefficient significantly affects the model outputs. In addition, the equations of the Swale Drainage Model predict the drainage flow behavior of the swale accurately. This indicates that the exponential functions in the Swale Drainage Model are appropriately developed and represent the drainage patterns.

The existing models presented in the previous studies have different shortcomings when compared to the model presented in this study. In EPA SWMM, which is commonly used in modeling of vegetated swale, specific inputs for defining soil, storage, and drainage properties are missing, which leads to missing results regarding drainage and storage in the swale. However, the model presented here explicitly takes into account storage and drainage layers while calculating flow rates. HEC‐HMS and SWAT integrate various LID practices into the watershed models by assigning specific land cover types in sub‐catchments and employing curve numbers. However, these models lack dedicated modules for simulating LID, limiting their flexibility in capturing the nuanced hydrological behavior of LID systems.

In the model presented by Grinden (2014), the model was not validated with experimental data, whereas the model presented here is validated by the RWS experimental results. The model developed by Chen et al. (2017) was calibrated using experimental data; however, the calibration results were not as satisfactory as the ones presented in this study. Moreover, their model did not consider drainage in swales, which is incorporated in the model presented in this study. Consequently, the model presented in this study represents a step forward in addressing the limitations of the existing models. However, there is still potential for further refinement and improvement.

## CONCLUSIONS

In this study, an integrated hydrological model, which consists of two modules, that is, the “Swale Overflow Model” and the “Swale Drainage Model,” has been developed to calculate both the overflow and the drainage flow of a vegetated swale. The measured data obtained from the experimental setup called RWS was employed in developing the model. The equations of the model were solved using MATLAB, and the performance of the model representing the hydrological behavior of a vegetated swale was shown. The Swale Overflow Model employs the Kinematic Wave Theory in determining the flow over the swale surface. It takes into account the storage capacity of the swale and also incorporates the output of the Swale Drainage Model, that is, drainage flow through the swale soil. The Swale Drainage Model involves two distinct exponential functions to represent the rising and falling limbs of the drainage flow hydrographs.

The peak drainage flow rate was significantly affected by rainfall intensity, rainfall duration, and areal rainfall. Therefore, an empirical equation puts forward the relationship between the peak drainage flow and rainfall intensity, rainfall duration, and the effective watershed area. The experimental results from the RWS System (Saraçoğlu & Kazezyılmaz‐Alhan, 2023) were compared with the hydrological model results, and goodness of fit measures were calculated. The results indicate a very good performance of the developed model in representing the hydrological behavior of swales. Consequently, the developed model provides an efficient tool that can be used for practical purposes in the design process of vegetated swales for sustainable urban stormwater management solutions.

Further studies will include testing the accuracy and capability of the model with a real swale implementation in an urban area under real and extreme storm events. In addition, this model can be incorporated into existing software, for instance, EPA SWMM, to improve the shortcomings of swale modeling capabilities.

## AUTHOR CONTRIBUTIONS

**Kebir Emre Saraçoğlu:** Conceptualization; methodology; software; formal analysis; validation; writing—original draft; writing—review and editing. **Cevza Melek Kazezyılmaz‐Alhan:** Conceptualization; methodology; software; supervision; formal analysis; validation; writing—original draft; writing—review and editing; funding acquisition.

## CONFLICT OF INTEREST STATEMENT

The authors declare no conflict of interest.

## Data Availability

All data, models, and code, generated or used during the study, appear in the submitted article, and some or all data, models, or code that support the findings of this study are available from the corresponding author upon reasonable request.
